# Differentiating ADHD from oral language difficulties in children: role of movements and effects of stimulant medication

**DOI:** 10.1186/s12888-014-0370-0

**Published:** 2014-12-31

**Authors:** Carroll W Hughes, Joyce Pickering, Kristi Baker, Gina Bolanos, Cheryl Silver

**Affiliations:** Department of Psychiatry, University of Texas Southwestern Medical Center, 5323 Harry Hines Boulevard, Dallas, 75390-9044 Texas USA; Department of Rehabilitation Sciences, University of Texas Southwestern Medical Center, Dallas, Texas USA; Shelton School, Richardson, Texas USA

**Keywords:** Oral language disorders, ADHD, Inattention, Hyperactivity/Impulsivity, Language, Cognitive processing, Continuous performance testing, Movement detection

## Abstract

**Background:**

The current study was designed to test if an objective measure of both attention and movement would differentiate children with Oral Language Disorders (OLD) from those with comorbid Attention Deficit/Hyperactivity Disorder (ADHD) and if stimulant medication improved performance when both disorders were present.

**Methods:**

The sample consisted of thirty-three children with an identified oral language disorder (of which 22 had comorbid ADHD) ages 6 to 13 who were enrolled in a yearlong intensive learning intervention program. Those on a stimulant medication were tested at baseline and again a year later on and off medication.

**Results:**

Objective measures that included an infrared motion analysis system which tracked and recorded subtle movements discriminated children with OLD from those with a comorbid ADHD disorder whereas classic attention measures did not. There were better attention scores and fewer movements in children while on-medication.

**Conclusions:**

Use of an objective measurement that includes movement detection improves objective diagnostic differential for OLD and ADHD and provides quantifiable changes in performance related to medication for both OLD and ADHD.

## Background

A language impairment (LI) or language disorder is a deficit or delay in receptive language (the understanding of spoken language by others) and/or expressive language (the sharing of thoughts, ideas, and feelings). An assortment of terms have been used to refer to this LI condition, including specific language impairment (SLI), developmental language disorder, expressive language disorder, developmental dysphasia or aphasia [1], and various subtypes of communication disorders [2]. Attention-Deficit/Hyperactivity Disorder (ADHD) is one of the most common comorbid diagnoses for children with an expressive language disorder or mixed receptive-expressive language disorder (referred to in rest of article as oral language difficulties – OLD) [3,4]. Conversely, studies have indicated that children with ADHD are at risk for learning problems or learning disorders [5,6]. An estimated 50% of children with ADHD have a comorbid oral language deficit [4,7], while 20 to 60% of children with ADHD have one or more learning disabilities or language problems [8]. Children with co-occurring OLD and ADHD are expected to experience more academic difficulties [9] and a wide variety of performance issues in language, coordination, attention, and perception, and as well have more difficulties with social skills and emotional well-being [10].

Due to inattention and impulsivity in children with OLD, they are frequently mistaken for symptoms of ADHD. Studies have found that children with deficits in pragmatic language displayed excessive talking, provided insufficient information upon responding, had poor turn-taking skills, and demonstrated difficulty maintaining topics and staying on task [11,12]. Furthermore, in a study by McInnes and colleagues [13], lower listening comprehension and working memory performance was evident in children with language impairment. The diagnostic dilemma becomes that these inattention symptoms and behavior control difficulties in children with OLD can appear quite similar to inattention and hyperactivity symptoms seen in children with ADHD and consequently, some with OLD only may be prescribed medication without established efficacy. Or, the language disorder may be missed all together and the focus becomes ADHD without addressing the language disorder. And then there are those with OLD and comorbid ADHD where an accurate language disorder diagnosis fails to identify the concomitant ADHD where the addition of a medication intervention may prove beneficial.

Due to overlapping symptoms and similar behavior disturbance, differential diagnostic accuracy can be challenging. It is important to establish an accurate diagnostic differential to guide appropriate treatment intervention (e.g., whether one should be tried on a stimulant medication). There is a need for an objective measure to improve differential diagnostic acumen for co-occurring OLD and ADHD as a precursor to medication intervention. Medication is one of the most common approaches to treat ADHD symptoms in children and has been successfully associated with improvements in functioning [14]. A myriad of studies have identified stimulant medication to improve executive functioning in adults and children with ADHD, while other studies have shown stimulant medication to improve behavioral issues, such as self-regulation related to movement [14,15]. Little research exists for those with a well-defined diagnosis of concomitant OLD/ADHD.

Fortunately, an objective measure of attention such as the Continuous Performance Test (CPT) has proven an effective, popular, and efficient means of objectively assessing attention [16], especially in individuals with suspected ADHD [17]. The CPT minimizes any aspect of potential bias from self, parent, teacher, or clinician reporting of symptoms. The mechanism by which a CPT detects ADHD-like symptoms is by identifying the test-takers’ response patterns. Inattentive and hyperactive/impulsive symptoms are two separate variables, tabulated by the amount of missed responses to “target” stimuli (omissions) and responses to “non-target” stimuli (commissions) compared to a normative sample. Errors of omission are assumed to reflect symptoms of inattention and commissions hyperactivity/impulsivity [18-20]. Most studies examining the psychometrics of various CPTs agree that it is a reliable and valid clinical tool to use as part of a more comprehensive assessment of ADHD [21] but not OLD. However, the CPT alone has not been established as a tool for “diagnosing” ADHD. There is insufficient data in the extant literature to demonstrate the CPT’s ability to differentiate OLD from OLD with ADHD. One explanation for that is the traditional CPT utilizes only measures of attention which is a common deficit found for both disorders and does not include movement variables [22-24].

The Quotient® ADHD System is a classic objective measure of various CPT go, no-go, attention variables (e.g., accuracy, omissions, commissions, variability), but unlike other CPTs, it is the only one combined with infrared motion detectors (e.g., number of movements, immobility duration, displacement, area) [Quotient®, BioBehavioral Diagnostics, Inc., 2010; purchased by Pearson, Inc., 2013] [25]. Previous work with motion detectors has demonstrated that children with ADHD in a CPT task spent 66% less time immobile than normal children, moved their head 3.4 times as far, covered a 3.8 times greater area, and had a movement pattern that was more complex [26]. Teicher’s research group [24] has recently suggested that the inability to inhibit subtle movements detected by the Quotient® for individuals with ADHD may represent deficits in functional activity of the cerebellar vermis, whereas the inhibition of attention measures may reflect deficits in functional capacity of frontostriatal areas and that both areas respond positively to stimulant medication.

The study reported here tested both attention and movement measurements in children with an OLD and those with both disorders (OLD/ADHD). Additionally, since research supports that stimulant medication improves attention and body control, we assessed the effects of being on- versus off- medication differences for OLD/ADHD at baseline and following focused training for language impairment again a year later where individuals had been on stimulant medication at both time points. An initial dissertation study at baseline had hypothesized that both attention and movement scores would differentiate OLD from OLD/ADHD but only movement was found to discriminate the two (30). Based on that finding, for the longitudinal follow-up study a year later, it was hypothesized that only movement measures would differentiate those with OLD from a group with a comorbid OLD/ADHD diagnosis (31). It was further hypothesized that the effects of stimulant medication treatment would improve attention scores for those with ADHD, while decreasing movement, and that these effects could be seen at baseline and replicated a year later.

## Methods

### Participants

**Figure 1:**
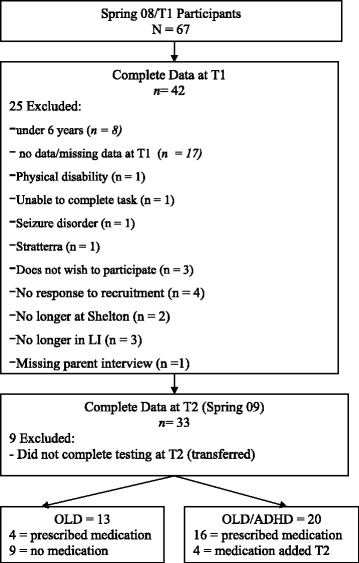
**Participant data collection flow chart.**

Thirty-three participants were between the ages of 6 and 13 years (15 = male; 18 = female) from an original sample of 67. The participants in the study attended a specially designed early language intervention program at the Shelton School [27], a large private school for children with learning differences, in Dallas, Texas. To be included in the study, the participant had to have a primary diagnosis of Oral Language Disability (OLD) diagnosed by a team of speech-language pathologists, licensed psychologists, and educational diagnosticians at Shelton School and enrolled in the school’s specially designed early intervention program. OLD children had: (a) low average (85 – 89) or below average (<85) verbal IQ, (b) below average (<85) auditory processing, processing speed, visual perceptual ability, reading comprehension, spelling, or handwriting, and (c) average reading rate and accuracy (85 – 115). In addition, their receptive and expressive language performance was in the moderate to severe range of impairment or below 85. Those participants with a comorbid ADHD diagnosis had one of the four subtypes of Predominantly Inattentive Type, Predominantly Hyperactive-Impulsive Type, Combined Type, or ADHD Not Otherwise Specified. Participants in the study were not excluded for taking a stimulant ADHD medication, but were asked to be able to be tested on and off their medication (i.e., parent and child provided consent and assent). The choice to be on medication was based on parent and community physician decisions for treatment. School and study personnel had no control over that, rather we could only indicate if they were on the medication or not. The school nurse knew what medication they were taking, but not the doses. All testing was done within the first two hours of the school day. One on non-stimulant psychotropic medication was excluded. Participants with a history of head injury (a period of unconsciousness followed by lasting impairment) or a neurological disorder, such as a seizure disorder, were excluded (Figure 1).

To determine if the child had ADHD, the parent and the child separately were administered the Kiddie Schedule for Affective Disorders and Schizophrenia in School Age Children, Present and Lifetime (K-SADS-P/L) [28] which uses the same diagnostic criteria as the DSM-IV-TR. All items of ADHD syndrome were administered to both the parent and the child. Specifically, to be diagnosed with ADHD, a participant had to receive ratings of “three - definitely present” for six of the nine inattentive symptoms (ADHD-IA), or six of the nine hyperactive symptoms (ADHD-HI), or a minimum of six inattentive and six hyperactive/impulsive symptoms (ADHD-C) prior to the age of seven. Children were diagnosed with ADHD NOS if ratings did not meet full symptom criteria (e.g., a minimum of 6 criterion symptoms rated as a “three”), but had prominent symptoms of inattention and/or hyperactivity/impulsivity present and causing impairment (e.g., a minimum of four symptoms but < 6 criterion symptoms rated as a “three”). Reliability studies of the K-SADS-P/L report high inter-rater reliability (range: 93% to 100%) and excellent test-retest reliability [28].

### Measures

#### Quotient® ADHD System

The Quotient® is a computerized continuous performance test assessing a participant’s ability to pay attention and ability to sit still [25]. It provides precise, reliable, and reproducible information on symptom severity under controlled conditions that replicate a classroom environment. Participants wear a headband with an attached motion reflector, which faces an infrared motion analysis tracking system (located just above the computer monitor). Movement greater than 0.04 mm resolution is detected by the tracking system from the motion reflector, and movement data is collected nearly 50 times per second throughout the 15-minute task [24]. Participants are asked to press the space bar each time they see an eight-pointed star but to inhibit their response to five-pointed stars. Each target appears on a white computer screen for 200 milliseconds in different, random spots on the screen at 2-second intervals. Variables collected include movement (number of position changes), displacement (total distance moved), temporal scaling (pattern of movement in time; lower values reflect less movement), accuracy (percentage of correct responses), omission errors (percentage of missed targets), commission errors (percentage of inaccurate responses to non-targets), latency (average amount of time to respond correctly), and variability (variation in response time to the correct target). We recently confirmed the factor loadings for ADHD using the Quotient® [29].

### Design and procedure

This study was reviewed and approved by the Shelton School Internal Review Board (IRB) and the University of Texas Southwestern Medical Center IRB. Both the child and parent gave informed verbal and written assent and consent to participate in the study.

Baseline data Time 1 (T1) data were collected during the Spring [30] and Time 2 (T2) data were collected again during the following Spring after completing a specially designed year-long oral language school intervention [31]. All testing was conducted at the Shelton School in a quiet, secluded testing area during the first periods of school in the morning. Participants were tested one at a time. During the 15 minute test, the administrator stayed in the testing room to notate observations, without talking to the participant and to assure that they remained on task. Participants off of their medication were tested after arriving at school, and then upon completion were taken to the school nurse for medication administration and then onto class. Testing was conducted on different days for those who participated in the either on or off of their medication condition (the counterbalanced order of testing was random). That they had taken, or not taken their medication prior to arriving to school, was verified by the study staff prior to testing. Upon completion of the test, data were transmitted and analyzed by BioBehavioral Diagnostics on a central server. Data were then compared to a normative group by age and gender and a report was produced including statistical and graphical information about attention and movement variables.

### Data analysis

Demographic characteristics for the overall sample are described using the sample mean and standard deviation for continuous variables and the frequency and percentage for categorical variables. To test the Quotient® ADHD attention and movement variables, the basic design was a two-group (OLD vs. OLD/ADHD), by Time (T1 and T2) repeated measures Analysis of Variance (ANOVA) design using SPSS Version 19. A secondary analysis of medication (ON or OFF) by Time analysis was conducted for an OLD/ADHD subgroup of 16 participants taking stimulant medication at both time points (the four OLD participants on medication were excluded for the medication analyses).

## Results

Of the sixty-seven participants enrolled in the Shelton School Language Intervention program, thirty-three met criteria for the current study (see Consort Figure 1). There were 18 females and 15 males, ranging in age from 6 to 13 (*M* =9.4 years, *SD* = 2.1) who completed all testing. All had an OLD diagnosis and 22 (62.9%) also met criteria for a diagnosis of ADHD. A separate secondary analysis was performed on all participants who were also taking a stimulant medication at both time points on and off of medication (Table 1).

**Table 1:** **Demographic characteristics of the study’s sample**

	**Total sample**	**OLD**	**OLD/ADHD**
	**(N = 33)**	**(n = 13)**	**(n = 20)**
	**N (%)**	**n (%)**	**n (%)**
**Gender**			
Male	15 (46%)	7 (54%)	8 (40%)
Female	18 (54%)	6 (46%)	12 (60%)
**Ethnicity**			
Caucasian	27 (77%)	11 (85%)	15 (75%)
Hispanic	3 (9%)	0 (0%)	2 (10%)
African			
American	1 (3%)	0 (0%)	1 (5%)
Asian	1 (3%)	0 (0%)	1 (5%)
Other	3 (8%)	2 (15%)	1 (5%)
**ADHD type**			
Inattentive	6 (18%)	N/A	6 (30%)
Hyperactive-			
Impulsive	1 (3%)	N/A	1 (5%)
Combined	6 (18%)	N/A	6 (30%)
NOS	7 (22%)	N/A	7 (35%)
^a^Medication	20 (61%)	4 (31%)	16 (80%)

^a^Participants prescribed stimulant medication duration of study period.

To assess if the OLD and OLD/ADHD groups were equivalent in overall functioning and severity with regard to language and cognitive functioning, independent-samples *t* tests indicated no group differences with respect to language functioning (Clinical Evaluation of Language Fundamentals – CELF), verbal cognitive ability (Slosson Intelligence Test-Revised), and nonverbal cognitive ability (Wechsler Nonverbal Scale of Ability). Teacher reports on the Behavioral Assessment System for Children (BASC) were examined to determine if groups differed with respect to internalizing symptoms (Internalizing Composite), externalizing symptoms (Externalizing Composite), overall behavioral symptoms (Behavioral Symptoms Composite) and adaptive behavior (Adaptability Composite). No differences were found and hence, none of these measures were used as covariates for the analyses that follow.

### Movement differences by diagnostic groups

The means and standard deviations for the various Quotient® movement variables are reported in Table 2. The OLD/ADHD group had a significantly greater number of movements at both Time 1 and Time 2 than the OLD group *F*(1,29) = 8.54, p < .007, η^2^ = 0.23, a large effect size. Effect size is a partial eta-squared (η^2^) where values of .01, .06, and .14 represent small, medium, and large effects sizes [32]. Likewise, children with OLD/ADHD had significantly greater distance in their movements (“displacement”) at both Time 1 and Time 2 than the OLD group *F*(1,29) = 7.76, p < .01, η^2^ = 0.21) and greater area represented in their movements at both Time 1 and Time 2 than the OLD group *F*(1,29) = 10.64, p < .003, η^2^ = 0.27. Finally, children with OLD/ADHD had a higher frequency of movements (“temporal scaling”) at both T1 and T2 than the OLD group *F*(1,29) = 8.30, p < .01, η^2^ = 0.22, but the spatial complexity of the movements did not differ between groups. There were no interactions or effect of T1 versus T2. The test-retest reliability for the movement measures was good to excellent with all p values less than 0.001: immobility (*r* = .75), movement (*r* = .82), displacement (*r* = .75); area (*r* = .66); spatial complexity (*r* = .65) and temporal scaling (*r* = .86).

**Table 2:** **Two-way repeated measures analyses of variance to examine Quotient® movement variables for diagnostic groups**

	**OLD (n =12)**		**OLD/ADHD (n = 19)**						
	***M (SD)***		***M (SD)***						
	**T1**	**T2**	**T1**	**T2**	**Statistic**		**Value**	***p***	**η** ^**2**^
Immobility duration	213.58(116.84)	222.25 (159.82)	115.11 (122.70)	118.32 (110.82)	Group	*F(1,29)*	5.49	.26	
Movements	2032.67(1020.75)	2458.25 (2086.34)	4837.84 (2746.45)	4457.68 (2646.04)	Group	*F(1,29)*	8.54	.01*	.23
Displacement	2.73 (1.58)	3.82 (4.22)	8.70 (6.15)	8.11 (6.61)	Group	*F(1,29)*	7.76	.01*	.21
Area	66.17 (50.41)	107.67 (153.53)	265.37 (154.55)	266.11 (229.09)	Group	*F(1,29)*	10.64	.003*	.27
Spatial complexity	1.20 (.10)	1.20 (.13)	1.12 (.14)	1.11 (.14)	Group	*F(1,29)*	0.10	.09	
Temporal scaling	.60 (.24)	.59 (.37)	.98 (.42)	.98 (.42)	Group	*F(1,29)*	8.30	.01*	.22

*Significant *p-value* alpha level of .01. η^2^ effect sizes of .01, 06, and .14 represent small, medium, and large effects sizes respectively.

Immobility Duration: Average amount of time, in seconds, spent sitting still (moving less than 1 mm) during a 5 min. period.

Movements: Average number of position changes (movement greater than 1 mm), measured in total meters during a 5 min. period.

Displacement: Total distance traveled (in meters) by the marker during a 5 min. period.

Area: Size and shape, measured in cm^2^, of the space covered by the marker during a 5 min. period.

Spatial Complexity: Complexity of the movement path. (Values range from one to two). Lower values indicate more linear, back & forth movement; higher values indicate more complex movement.

Temporal Scaling: Frequency of movement (scale from 0 to 1; 0 = no movement and 1 = constant movement).

A MANOVA at baseline to minimize possibility of Type I error, found no significant group differences on the attention variables so they are not reported, Wilk’s Λ = 0.915, F(4, 46) = 1.07, p = .38, [30], or again at T2 [32]. The attention scores were impaired compared to norms (e.g., mean accuracy scores for OLD of 81% and 78% for OLD/ADHD whereas normal performance would be in the low to mid 90s [23]. Separate power analyses indicated that much larger group sizes would be required (>200) for differences at p < .05 and power of 80.

A discriminant function analysis was conducted using all of the baseline movement scores to predict OLD versus OLD/ADHD group membership. The Wilk’s Lambda chi square was significant at 16.3 (df = 6), and p = .01, with 82.8% of the groups correctly classified. The Kappa was 0.66, p < .001. A separate sensitivity/specificity analysis indicated 73.3% sensitivity and 92.9 specificity, with a positive predictive value of 91.7%. The findings provide strong support that body movements as measured by the Quotient® during a CPT task can significantly differentiate those children with OLD only diagnoses from those with OLD and ADHD and are stable indices whereas by contrast, there were no differences based on classic CPT attention measures.

### Movement differences by medication condition: a secondary analytic approach

To assess the effects of medication, a secondary set of analyses utilized a within subject two group (On Medication versus Off Medication) repeated measures ANOVA based on Time 1 and Time 2 (a year later) for a subgroup who were on medication at both time periods. The means and standard deviations for the various Quotient® ADHD System movement variables are reported in Table 3. All movement measurements were significantly different when on medications. There were no interactions or effect of T1 versus T2. Children spent more time sitting still (“immobility duration”) compared to their performance off medication at both T1 and T2 *F*(1,15) = 20.54, p < .00, η^2^ = .58, representing a large effect size. They had fewer position changes (less “movement”) when tested on medication compared to their performance off medication at both T1 and T2 *F*(1,15) = 26.10 p < .00, η^2^ = .64, and shorter total distance of movements (“area”) when tested off medication *F*(1,15) = 22.45 p < .00, η^2^ = .60. Children had a lower “area” score (i.e., the space they moved in was smaller) when tested on medication compared to their performance off medication at both T1 and T2 *F*(1,15) = 31.08 p < .00, η^2^ = .67. They also had a higher “spatial complexity” score (i.e., movements were qualitatively more complex) when tested on medication compared to their performance off medication at both T1 and T2 *F*(1,15) = 17.54, p < .00, η^2^ = .54. Children tested on medication had significantly less frequent movements (“temporal scaling”) at both T1 and T2 compared to more frequent movements when tested off medication *F*(1,15) = 24.49 p < .00, η^2^ = .62. Using the Quotient® ADHD System to measure movement, children tested on medication spent significantly more time sitting still, had fewer position changes, traveled less distance in their movements, had smaller area of movement, had less frequent movements, and had more complex movements at both T1 and T2.

**Table 3:** **Two-way repeated measures analyses of variance to examine Quotient® movement variables for medication condition**

	**Off medication (n = 16)**		**On medication (n =16)**						
	***M (SD)***		***M (SD)***						
	**T1**	**T2**	**T1**	**T2**	**Statistic**		**Value**	***p***	**η** ^**2**^
Immobility duration	123.38 (129.02)	106.81 (106.56)	195.56 (130.51)	244.50 (189.01)	Group	*F(1,15)*	20.54	.000*	.58
Movements	4841.56 (3007.52)	4916.62 (2768.34)	2342.44 (1409.24)	2343.56 (1930.90)	Group	*F(1,15)*	26.10	.000*	.64
Displacement	8.83 (6.76)	9.23 (7.06)	3.44 (2.64)	3.54 (3.57)	Group	*F(1,15)*	22.45	.000*	.60
Area	250.25 (172.73)	300.13 (255.54)	87.25 (84.52)	91.06 (113.39)	Group	*F(1,15)*	31.08	.000*	.67
Spatial complexity	1.14 (.15)	1.10 (.12)	1.19 (.14)	1.25 (.19)	Group	*F(1,15)*	17.54	.001*	.54
Temporal scaling	.99 (.44)	1.07 (.42)	.67 (.30)	.65 (.39)	Group	*F(1,15)*	24.49	.000*	.62

*Significant *p-value* alpha level of .01. η^2^ effect sizes of .01, 06, and .14 represent small, medium, and large effects sizes respectively.

Immobility Duration: Average amount of time, in seconds, spent sitting still (moving less than 1 mm) during a 5 min. period.

Movements: Average number of position changes (movement greater than 1 mm), measured in total meters during a 5 min. period.

Displacement: Total distance traveled (in meters) by the marker during a 5 min. period.

Area: Size and shape, measured in cm^2^, of the space covered by the marker during a 5 min. period.

Spatial Complexity: Complexity of the movement path (values range from one to two). Lower values indicate more linear, back & forth movement; higher values indicate more complex movement.

Temporal Scaling: Frequency of movement (scale from 0 to 1; 0 = no movement and 1 = constant movement).

### Attention differences by medication condition

The means and standard deviations for the various Quotient® ADHD System attention variables are reported in Table 4. Children had better “accuracy” scores at both time points while medicated *F*(1,15) = 8.14, p < .01, η^2^ = .35. Likewise they had fewer “omissions” (i.e., fewer missed targets) when tested on medication compared to their performance off medication *F*(1,15) = 9.90, p < .01, η^2^ = .40, but no differences for incorrect responses to non-targets (“commissions”). “Variability” (defined as the standard deviation of response time to target) was significantly lower on medication compared to their performance off medication *F*(1,15) = 33.62, p < .00, η^2^ = .69. For “latency”, children responded significantly faster when they were on medication compared to their performance off medication *F*(1,15) = 28.03, p < .00, η^2^ = .65. For Coefficient of Variance (COV) [a more stringent measure of response consistency: (100 x variability)/latency], response consistency was significantly greater when children were tested on medication compared to their performance off medication *F*(1,15) = 32.28, p < .00, η^2^ = .68. These attention scores clearly indicate that stimulant medication improves attention performance during a fifteen minute continuance performance task with significantly better accuracy, fewer omission errors, faster response time, and less variability.

**Table 4:** **Two-way repeated measures analyses of variance to examine Quotient® attention variables for medication condition**

	**Off medication (n = 16)**		**On medication (n =16)**						
	***M (SD)***		***M (SD)***						
	**T1**	**T2**	**T1**	**T2**	**Statistic**		**Value**	***p***	**η** ^**2**^
Accuracy	81.14 (13.81)	85.91 (12.09)	86.44 (12.40)	89.83 (6.30)	Group	*F(1,15)*	8.14	.01*	.35
Omission errors	14.19 (14.49)	11.11 (15.11)	4.08 (5.88)	3.89 (5.37)	Group	*F(1,15)*	9.90	.01*	.40
Commission errors	23.56 (16.27)	16.96 (13.33)	23.14 (19.74)	16.46 (8.94)	Group	*F(1,15)*	.06	.81	
Variability	217.44 (81.27)	198.00 (88.15)	153.63 (76.77)	122.75 (55.26)	Group	*F(1,15)*	33.62	.00*	.69
Latency	598.37 (105.77)	584.63 (89.17)	545.44 (81.50)	495.37 (80.31)	Group	*F(1,15)*	28.03	.00*	.65
COV	35.50 (11.42)	32.25 (10.06)	27.13 (10.91)	23.69 (7.49)	Group	*F(1,15)*	32.28	.00*	.68

*Significant *p-value* alpha level of .01. η^2^ effect sizes of .01, 06, and .14 represent small, medium, and large effects sizes respectively.

Accuracy: Percentage of correct responses to both target and non-target.

Omission Errors: Percentage of missed targets.

Commission Errors: Percentage of incorrect responses to non-target.

Latency: Mean time, in milliseconds, to respond to target.

Variability: Standard deviation of response time to target.

Coefficient of Variance (COV): A more stringent measure of response consistency: (100 x variability)/latency.

### Attention state differences by medication condition

Additional measures calculated by the Quotient® ADHD System are known as attention state variables. For example, attention shifts and the number of shifts in attention state are defined as On Task (percent of time hit many targets and few non-targets), Distracted (percent of time hits some targets and some non-targets; accuracy is better than chance), Impulsive (percent of time hits many targets and some non-targets), Random Responding (hits most targets and non-targets; accuracy of responding is as good as chance), Minimal Responding (misses most targets and non-targets; accuracy is about as good as chance), and Contrary (response accuracy is significantly worse than chance). The analyses of these variables indicate that the children spent more time responding “on task” when they were tested on medication *F*(1,15) = 4.66, p < .05, η^2^ = .24, spent a lower percentage of time in a “distracted” attention state, and less time in the “minimal responding” attention state profile (i.e., children were less likely to make both omission and commission errors) *F*(1,15) = 4.92, *p* < .04, η^2^ = .25.

## Discussion and conclusions

This study found that children with oral language disorder (OLD) and comorbid ADHD performed significantly different from those with an OLD-only diagnosis based on subtle, measureable movement scores but not classic CPT attention variables. Importantly, movement measures such as position changes, larger displacement of head movements (i.e., total distance traveled per unit time), larger area of movement, and more temporal scaling (i.e., frequency of movement) as measured by the Quotient® ADHD System do significantly differentiate the two groups with good statistical group classification and sensitivity and specificity. This suggests that relying on CPT-like measures alone (without movement measurements) would probably not meaningfully differentiate the two groups. Unlike other CPTs that do not measure movement, these additional movement variables are valuable additions to tests to distinguish the overlap of core ADHD symptoms in children with OLD and would assist diagnostic classification. These findings lend support to Ohashi et al.’s [24] contention that the inability to inhibit subtle movements detected by the Quotient® for individuals with ADHD may represent deficits in functional activity of the cerebellar vermis [33] that can uniquely differentiate children with OLD/ADHD from those only with OLD. Both groups appeared to equally show impairment in measures of attention which likely reflect deficits in functional capacity of frontostriatal areas [34,35]. The Quotient® significantly improves the ability to accurately differentiate OLD from OLD/ADHD children when compared to traditional CPT attention measures only. Utilizing assessments that included objective, quantifiable movement measures may improve the accuracy of the differential diagnosis.

CPTs have proven to be a reliable and valid clinical tool to include as part of a clinical assessment battery for ADHD but not for differentiating ADHD from OLD and learning disabilities in general, as both disorders have similar difficulty with various attention variables [18,20,21,36]. Unlike traditional CPTs, the Quotient® is a CPT designed to measure additional core symptoms of ADHD based on movement and attention shifts. In 2009, Baker found five movement variables to discriminate children with OLD from OLD/ADHD. The current study replicates the findings and extends them to repeated measures one year later supporting good test-retest reliability [30]. Specifically, four of the six Quotient® movement variables consistently discriminated children with OLD from OLD/ADHD. It discriminated all six movement measures when controlling for medication. Children with OLD/ADHD did not differ from OLD children on immobility duration (i.e., time spent sitting still) or spatial complexity (i.e., complexity of movement path; lower value indicating more linear or back and forth movements). Despite questionable ADHD symptom overlap in OLD and ADHD children due to the use of subjective assessment protocols, this is the first study to investigate the utility of the objective Quotient® for discriminating ADHD symptoms between those children with OLD versus both OLD and ADHD. Findings suggest that an instrument which includes objective measurement of subtle movement could be a useful tool in the assessment of children with and OLD and those with OLD and comorbid ADHD.

While research suggests that stimulant medication improves attention and body control, the role of medication on attention and movement in children with OLD and ADHD has not been explored in terms of 1) does medication improve attention and/or movement, and 2) does medication improve attention and/or movement over time. By testing a subsample of the children who were on prescribed medication on separate days both on and off of their medications, and repeating the procedure a year later, individuals with both OLD and ADHD were found to have better body control (all six movement variables) when on stimulant medication and that the findings were stable over time. The current study’s findings were consistent with other studies identifying stimulant medication to improve body control in children with ADHD [14,15,37]. Equally important, results indicated that stimulant medication did improve overall attention performance in those with comorbid OLD and ADHD disorders. Specifically those children with both OLD and ADHD had significantly better accuracy, fewer omission errors, better response consistency, faster responding, and less variability when tested on medication compared to their performance off medication. The current study did not find differences between on versus off medication for commission errors (i.e., inhibition in responding to non-targets).

The current study also investigated the role of medication (on medication versus off medication) on attention state in OLD and ADHD children using the Quotient® ADHD System. It was expected that children tested on medication would have fewer attention shifts and more on task, less distracted, less impulsive, less random, less minimal, and less contrary attending. Results indicated that medication improved remaining on task (i.e., mostly responding accurately), being less distracted (i.e., less time hitting some targets and some non-targets), and minimal response patterns (i.e., less time missing most targets and non-targets). However, there were no significant differences in on versus off medication performance on attention shifts, impulsive responding, random responding, and contrary attention states.

### Methodological limitation and future research

Despite significant findings for differentiating diagnostic categories and effects of stimulant medication as identified by the Quotient® ADHD System, some limitations for the current study are considered. As noted earlier, the small sample size may not have detected important group, time, and interaction differences. Power analyses for the attention measures suggested that a fairly large number of subjects would be required (typically > 200 per group). Nonetheless, for the movement measures it is important to note that a number of significant differences had large effects sizes supporting that the differences were large enough to be detected with small sample sizes which supports that the findings are nonetheless robust. Likewise, the findings were corroborated when replicated a year later.

A second limitation suggests caution in generalizing findings. Children in the study represented a sample of opportunity selected from a pool of participants enrolled in a specially developed language intervention program [27] at a large private school for children for learning differences. Nonetheless, the importance is the unique opportunity to study what would be considered a fairly large homogenous sample of children with well identified oral/language differences involved in a structured learning intervention program, a subgroup which also had comorbid ADHD. And finally, secondary analyses related to the effects of medication merit caution given the limitations inherent in the design.
